# Supplementary material for: Are carbon emissions trading and green financial instruments synergistic? -Comprehensive quantitative research based on content analysis
Source: PLoS One. 2024 Mar 7;19(3):e0298601. doi: 10.1371/journal.pone.0298601 (PMC10919601; doi:10.1371/journal.pone.0298601)
Supplement: S1 Data — (ZIP) [file pone.0298601.s001.zip › Data/地级市Z_F工作报告.zip!/░▓╗╒/┘±╓▌╩╨/└┤╫╘╣1⁄2╓┌║┼í╛┬φ┐╦╩2╛▌═°í┐_2016.docx]

各位代表：

现在，我代表市人民政府，向大会报告工作，请予审议，并请市政协委员和其他列席人员提出意见。

2015年和“十二五”时期工作回顾

过去一年，在省委、省政府和中共亳州市委的坚强领导下，我们紧紧依靠全市人民，全面贯彻落实党的十八大、十八届三中、四中、五中全会和习近平总书记系列重要讲话精神，坚持稳中求进工作总基调，主动适应经济发展新常态，攻坚克难，积极作为，圆满完成了市三届人大六次会议确定的全部目标任务。预计，全市地区生产总值950亿元，增长9%；财政收入130.1亿元，增长12.4%；固定资产投资770亿元，增长18.3%；社会消费品零售总额437亿元，增长12.5%；进出口总额4.95亿美元，增长34.1%；城镇常住居民人均可支配收入23100元、增长9.2%，农村常住居民人均可支配收入9950元、增长10.1%；城镇登记失业率控制在3.5%，人口自然增长率控制在8.8‰；全面完成节能减排、大气和水污染防治目标任务；全市经济稳中有进、稳中有好，社会大局和谐稳定。

（一）坚持精准施策，实体经济实现新发展。面对经济下行、外需不畅、政策调整和环境约束趋紧等诸多困难，我们精准施策应对，全力打好“组合拳”。着力破解资金难题。出台产业引导基金、财政“借转补”资金和财政事后奖补资金等政策措施，共投入3.5亿元。增设银行网点12个、融资担保机构1家，全年新增贷款141.1亿元、比上年多增23.1亿元，贷款余额达784.3亿元、增长21.9%；注资3亿元成立市第二担保公司，全市融资性担保、政策性担保机构担保放大倍数分别达到3.9倍和4.7倍，在保余额87.5亿元；建安集团获批融资额度245亿元，到位资金75.9亿元；创新搭建“我要贷款”平台，发放贷款10.2亿元；在全国首创“532”小额贷款保证保险模式，为小微企业、“三农”提供无抵押担保贷款7200万元。保险业保费总收入43.8亿元，增长17.8%。金融业保持较快发展，银行、保险、证券、担保、典当、小额贷款等业务规模不断扩大。着力破解土地制约。全年争取省政府批准各类用地指标4.27万亩，是2014年的3倍，其中建设用地增减挂钩指标1.28万亩、工矿废弃地复垦利用试点指标1.55万亩、新增建设用地计划指标1.44万亩。着力推进大众创业、万众创新。强化为企业服务网上平台建设，解决相关企业历史遗留用地问题798亩，联系用工2.2万人；举办地产品对接会9场次，达成购销协议金额31亿元。全市净增规模以上工业企业122家、总数达793家，净增限额以上商贸企业87家、总数达603家；新增私营企业8426户、增长43.3%，新增个体工商户28301户、增长27.5%。

（二）坚持协调发展，结构调整取得新进步。预计，三次产业结构调整为21：39.1：39.9，与上年相比，一产占比下降1个百分点，三产占比上升1.4个百分点。坚定不移地实施工业强市战略。全年工业投资305亿元、增长26%，规模工业增加值250.1亿元、增长11%。现代中药产业实现产值230亿元，增长11%；白酒产业实现产值82亿元、增长16%，其中古井集团白酒主业逆市上扬，实现经营收入51亿元、增长9.7%，荣获2015年度全国工业企业质量标杆奖。高新技术产业实现产值285亿元，增长14%；安徽亳州国家农业科技园区获科技部批准，安徽现代中药产业发展集聚基地成为全省首批14个战略性新兴产业集聚发展基地之一。新认定高新技术企业6家，新建院士工作站2个，新增授权专利1150件，新增省级新产品5个、省级企业技术中心2家。坚定不移地推动农业现代化。全年粮食总产97亿斤，蔬菜种植面积197万亩、其中设施蔬菜种植面积60万亩，中药材种植面积110万亩、其中标准化种植面积15万亩；新增农民合作社1504家、总数达6455家，新增家庭农场1079家、总数达2045家，规模养殖比重达80%，农业适度规模经营面积达342.3万亩；农机总动力867万千瓦、净增28万千瓦，利辛县被评为全国平安农机示范县；产值亿元以上农产品加工企业发展到107家、新增18家，全市规模以上农产品加工产值达590亿元，增长14.8%。市级农产品质量安全监测中心完成主体工程。全面推进小型水利工程改造提升工作，新建加固小水闸106座，改造中小灌区4万亩，改造末级渠系42.5万亩；2015年度中小河流、洼地治理和涵闸除险加固工程全面完工。完成粮食老危仓库建设项目88个，新建粮库75万吨。新建气象防灾减灾标准化乡镇和示范村各8个。全市大宗农作物种植品种保险参保面达90%，累计受益农户74.8万户。坚定不移地提升第三产业。全市旅游总收入达100亿元、增长12.6%，创建4A级旅游景区3个、三星级以上农家乐15家；新获评省级优秀旅游乡镇3个、旅游示范村8个；推出乡村旅游精品线路7条，拍摄美丽乡村之旅专题片24集。新改建社区菜市场14个、便利店48个、早餐店51个、家政服务网点8个，万达广场等11个市本级商贸流通项目完成投资61.4亿元。新建配送中心2个、新改建连锁网点160家，“新网工程”网络覆盖面不断扩大。筹建亳州青年创客空间，青年电商产业园入园企业达70余家；建成农业物联网示范点22家，阿里巴巴特色农产品“亳州馆”年销售额突破3亿元；药通网成功获批国家互联网药品第三方交易许可证。完成房地产开发投资205亿元，商品房销售面积359万平方米，房地产市场保持稳定。邮政和快递业务收入4.7亿元，增长27.1%。

（三）坚持双轮驱动，改革开放迈出新步伐。着力深化改革，不断扩大开放，为加快发展提供强大动力。行政管理体制改革不断深化。在全省率先完成权力清单、责任清单、行政收费清单和政府公共服务清单编制运行工作。整合卫生、计生职责，组建市、县区卫生计生委；整合市药监、工商、质监等部门食品药品监管职责，组建市食品药品监管局；组建市信息局；设立市数字城管指挥中心和市园林管理局；调整市扶贫开发办公室机构编制；理顺市高标准基本农田建设工作机制。加快推进商事制度改革，提前实现“三证合一”。全面完成公务用车制度改革。农村综合改革扎实推进。涡阳县全面完成农村土地承包经营权确权登记颁证工作，其他县区全面展开；小型水利工程管理体制改革加快推进，管护耕地面积达438万亩；涡阳县获批国家首批农村承包土地经营权抵押贷款试点。财税金融改革深入实施。结构性减税和普遍性降费政策全面落实，减免税费10.6亿元；“营改增”试点工作稳步推进；财政预决算和“三公”经费信息全部公开；盘活财政存量资金24.7亿元；新引进保险公司2家、证券机构1家；首发上市在审及辅导报备企业各1家，实现建市以来零的突破；新增“新三板”挂牌企业4家、区域股权交易中心挂牌企业18家。卫生教育等领域改革步伐加快。市、县两级公立医院综合改革和基层医改有序推进，蒙城县、谯城区乡村医生签约服务试点顺利实施，市人民医院药品零差率销售、医疗服务价格调整和药品耗材带量采购工作全面实施，县级公立医院药占比完成目标任务；国学经典教育纳入中考内容，蒙城县、利辛县被列为全省中小学教师无校籍管理试点县，亳州中药科技学校成为全国首批现代学徒制试点学校；市自来水公司完成“厂网分离”改革工作；建立居民用气、用水阶梯价格制度。开放型经济取得新进展。坚持把招商引资作为重中之重，大力实施靶标招商、精准招商，出台社会办医、办学、办养老机构和促进食品产业发展财政补助方案，规范招商引资项目流程，全年实际利用内资1231.9亿元，增长21.7%；实际利用外商直接投资6.6亿美元，增长10%；新签约招商引资项目236个、总投资994.9亿元，其中工业项目176个、总投资478.6亿元；成功举办第31届药博会和中国中医药发展大会，签约招商项目36个，总投资157.8亿元。新增进出口实绩企业22家，与115个国家和地区建立经贸往来关系。加快企业“走出去”步伐，设立境外营销机构2个。

（四）坚持项目带动，发展后劲得到新增强。坚持“经济工作项目化、项目工作责任化”，全力加快项目建设。完善项目推进机制。在落实项目法人责任制等8项制度的基础上，新出台政府投资项目管理等3个办法，加大“四督四保”工作力度，有力有效推动项目建设提速升级。全年谋划储备项目1100个，总投资3000亿元；列入省重点项目294个、增加24个，完成投资325亿元、增加20亿元；实施市重点管理项目385个、增加38个，完成投资505亿元、增加57亿元。突出抓好重大项目。投资6亿元的中联重机现代农机项目、投资8亿元的亳药堂药业项目、投资5亿元的鑫源实业20万套汽车零部件项目竣工投产，投资5亿元的修正健康饮品项目、投资15亿元的司尔特肥业、投资20亿元的九州方圆药业、投资19亿元的信利光伏电站、投资60亿元的利辛电厂等项目进展顺利、今年将陆续竣工投产；济祁高速永城至利辛段和省道307线改造工程亳州至蒙城段建成通车，商合杭高铁、北汽兴东方（蒙城）现代农业装备制造等项目开工建设，亳州机场选址初步确定，涡河高等级航道改造、引江济淮入亳工程等一批重大项目取得积极进展。着力解决项目难题。对历年未完成的重点建设项目、政府投资建设项目、社会资本投资类需政府协调解决问题的项目和新开工项目等4大类564个项目，实行清单管理，逐个进行调度，协调解决阻碍项目推进的问题2157个；其中市本级政府类项目完成投资63.8亿元，增长67.9%。

（五）坚持九城同创，城乡面貌呈现新变化。始终把“九城同创”作为重要抓手，统筹推进城乡规划建设管理，人居环境明显改善。突出规划引领。制定《亳州市城市控制性详细规划通则》。针对中心城区功能不全的问题，编制完成教育、卫生、水系沟通、菜市场和小吃摊点等27个专项规划。编制完成9个美丽集镇总体规划、31个美丽乡村规划。成功列为国家智慧城市试点。持续扩大投入。在中心城区实施城建项目162个、完成投资50.9亿元。全市新建高速公路91公里、干线公路325公里，改建农村公路436公里，改造危桥64座。220千伏漆园变电站建成投运，110千伏芍花变电站提前完工，农村电网改造完成投资3.5亿元。美丽乡村示范村和美丽集镇建设顺利推进。切实加强管理。坚持依法拆违控违，拆除违法建筑3.6万平方米。建立城区市政设施小修机制，2300余处（个）受损市政设施得到快速修复。开展主城区户外广告设施整治活动，依法拆除违规广告牌8886块。建成市主城区视频数据采集点5200个。数字城管应用系统完成6大类81个小类城市事件和37.4万多个城市部件精确确权工作并全面运行，为城市管理工作实现精细化和责任化奠定了基础。统筹推进县城规划建设管理六项行动、农村清洁工程、“三线三边”治理、“三治三增三提升”、“增绿增彩增果”行动，清理农村垃圾22.5万吨，清理河道5230公里。全面实施市中心城区“地净、路畅、水活、天蓝、林拥城”工程。加大城区道路清扫、冲洒频率，城市清洁度明显提升。352项道路交通拥堵综合治理任务已完成311项，投入道路征地拆迁资金16.4亿元，打通利辛路和建安南路“断头路”，涡河魏武大道桥顺利通车，改建后的文帝西路及桥、酒城大道下穿京九铁路立交桥建成通车，城区4个客运站平稳有序搬迁，13个道路交口渠化任务全面完成，35处交通标志线整改完毕，规范设置公交站台（牌）13处，新增出租车临时停靠点386个，搬迁主城区物流商户200多家，新建改造便民农贸市场7个，主城区道路拥堵状况明显缓解。“一涵四闸”水系贯通工程全面开工。加强秸秆综合利用，淘汰黄标车和老旧车9000余辆，淘汰改造10蒸吨以下燃煤锅炉174台（套），30家加油站完成油气回收改造，PM10平均浓度控制在每立方米87微克、下降9.4个百分点。扎实推进千万亩森林增长工程，完成人工造林14.5万亩；市植物园完成苗木栽培1800亩，开工建设“林拥城”环城林带，建设城市主干道和街头游园绿地326万平方米。蒙城县、利辛县荣获“国家园林县城”称号。

（六）坚持以人为本，人民生活有了新改善。31项民生工程投入资金107.6亿元，顺利完成年度任务。开工建设保障性安居工程2.9万套、基本建成1.5万套；改造农村危房8700套。新增城镇就业4.5万人、转移农村富余劳动力10.8万人。新增社区养老服务设施20所，新建、改扩建农村敬老院12所，农村五保集中供养能力达75%；城市低保标准由每人每月393元增加到432元，农村低保标准由每人每年2100元增加到2400元。96.6万农村居民和2.9万名学校师生的饮水安全问题得到解决。全面开展贫困人口精准识别工作，建立健全精准帮扶机制，实现脱贫8.9万人。发放各类助学资金1.1亿元、资助7.7万人次；全市高考本科达线率48.1%、高出全省近10个百分点；蒙城县成为全省唯一的第二批国家级农业职业教育和成人教育示范县；亳州师专升本工作获得全国院校设置评审委员会高票通过；义务教育均衡发展被教育部评为全国20个优秀工作案例之一。市人民医院新院二期工程、华佗中医院新院、市精神病医院等加快建设；全市新农合参合率达105.3%；新引进民营医疗机构6家，其中亳州药都肛肠医院建成并投入运营；建立国医大师传承工作室1个；创建市级卫生镇3个、卫生村12个；全市艾滋病病人抗病毒治疗覆盖率达95.6%。全市1372个市、县、乡、村公共文化场馆全部免费开放；87个乡镇（街道）综合文化站全部提标升级，建成13个农民文化乐园；开展各类文艺演出2570场，送戏进敬老院306场次，送电影下乡15096场次；全市广播镇通率达100%、自然村村通率达99%。深入开展精神文明创建活动，出台政策礼遇和帮扶道德模范、身边好人，全年5人新当选中国好人，10人新当选安徽好人，56人新当选亳州好人，2人获评为省第四届道德模范，10人获评为市第四届道德模范。国学经典进课堂被教育部列入社会主义核心价值观教育优秀案例。书香亳州建设有序推进，老庄文化研究不断深化。认真落实“三位一体”信访工作责任机制，全市到京非访、赴省越级访、来市上访人次分别下降44.8%、11.9%、36.5%。全市累计排查各类安全隐患8895处、增长22.8%，整改率达98.9%、提高1.8个百分点，省挂牌督办重大安全隐患整改如期完成，市挂牌督办重大安全隐患54处、已完成整改52处。全年化解农民工工资拖欠问题807起。不断强化食品药品安全监管，食品药品安全违法行为查处率达100%。扎实推进“平安亳州”建设，严厉打击各类违法犯罪活动，社会大局保持和谐稳定。市消防支队指挥中心、紫苑路消防站、涡阳县闸北消防站建成并投入使用，投入850万美元购置13辆消防车和一批消防器材装备，完成消防验收（备案）建设工程项目310个，监督服务和灭火救援水平有效提升。高度重视民族宗教工作，宗教教职人员生活津补贴由每月480元提高到1000元。在全市大力推广普及五禽戏，新增习练人员7万人；成功举办第二届中国（亳州）国际健身气功博览会，共有18个国家和地区的20支代表队来亳参会。顺利通过“六五”普法验收。市档案馆建成并投入使用。《亳州年鉴》荣获全国综合年鉴编纂出版质量评比二等奖。妇女儿童、老龄、慈善、红十字、残疾人等事业持续发展，关工委、外事、侨务、对台、供销、统计、地震、防灾减灾、应急管理和无线电管理工作取得新成绩。全民国防教育、国防动员、人民防空工作扎实推进，军民融合深度发展取得新进步。

（七）坚持“三严三实”，政府自身建设得到新加强。扎实开展“三严三实”专题教育，聚焦“忠诚、干净、担当”，着力解决“不严不实”问题。建立年度岗位责任目标和政府工作问题清单制度，逐一调度、加快推进。自觉接受人大法律监督，主动接受政协民主监督，建立定期联系人大、政协工作制度，办复人大代表议案5件、建议255件和政协委员提案327件，满意率均达97%以上。积极探索“互联网+政务服务”新模式，对政府权力实行“最小颗粒化管理”、细化为10305个单元，建成市县乡村四级网上办事大厅平台，累计受理116.2万件、办结109.8万件，群众满意率达99%以上；“我家亳州”APP正式运行，周活跃人数达10万余人（次）；建成市数据中心，整合市级44家单位、8亿多条数据信息，政务服务信息实现互联互通、共享共用。严格规范政府系统重大事项决策行为，完成重大事项合法性审查209件。着力推进审计监督，完成各类审计项目627项、比上年增加64项，全市政府性投资项目实现审计全覆盖。坚决执行中央八项规定和省委、市委三十条规定，全市“三公”经费下降7.6%。认真听取群众意见建议，整合“12345，电话找政府”市长热线职能，共接听群众电话9.7万多件，受理件办结率96%以上，群众满意率90%以上；举办“沟通——政务零距离”电视问政栏目10期，受理投诉举报147件、办结133件，办结率90.4%。

各位代表，2015年各项目标任务的完成，标志着“十二五”规划胜利收官。回顾过去五年，我们积极应对复杂局面，统筹做好稳增长、促改革、调结构、惠民生、防风险各项工作，全市经济社会进入了加速发展的崭新阶段。

——综合实力显著提升。经济总量连续迈上4个百亿元台阶、达950亿元，是“十一五”末的1.9倍，年均增长11%；人均地区生产总值由10615元增加到18900元，跨越3000美元大关；财政收入达130.1亿元，是“十一五”末的3.1倍，年均增长25.2%；社会消费品零售总额达437亿元，是“十一五”末的2倍，年均增长14.4%；实际利用外商直接投资6.6亿美元，是“十一五”末的4.2倍，年均增长33%。

——农业基础继续巩固。粮食生产连续迈上新台阶，先后成功创建全省首个“小麦亩产千斤市”和“吨粮市”，连续三年获“全国粮食生产先进单位”称号。农民合作社由1080家发展到6455家，是“十一五”末的6倍；农产品加工产值由180亿元增加到590亿元，是“十一五”末的3.3倍；市级以上农业产业化龙头企业由213家发展到466家，是“十一五”末的2.2倍。

——发展后劲明显增强。累计完成固定资产投资2721亿元，是“十一五”期间的3.7倍；累计完成工业投资1090亿元，是“十一五”期间的3.8倍；市场主体总数达到21.4万个，比“十一五”末净增10.2万个；规模以上工业企业达到793家，比“十一五”末净增545家；限额以上商贸企业603家，比“十一五”末净增499家。

——城镇建设步伐加快。城镇化率达37%，“十二五”期间年均提高1.6个百分点，年均增速居全省第1位。市主城区规划面积扩大到196平方公里，集聚人口52万人；三县县城建成区面积扩大到89平方公里，集聚人口75万人。全市开发区建成区面积由15.2平方公里拓展到84平方公里，是“十一五”末的5.5倍。

——人民生活日益改善。城镇居民人均可支配收入由15538元提高到23100元；农村常住居民人均可支配收入由4689元提高到9950元。城乡居民人均储蓄存款余额由6135元提高到11710元。城市居民人均居住面积由35.9平方米提高到42.7平方米；农村居民人均居住面积由32.8平方米提高到41.6平方米。私人汽车拥有量由15.7万辆增加到24.7万辆。有效解决422万农村居民和18.8万学校师生饮水安全问题；53万人稳定脱贫。

五年来取得的辉煌成绩，为加快活力亳州、美丽亳州、幸福亳州现代化建设奠定了坚实基础。这些成绩的取得，是省委、省政府和市委正确领导的结果，是市人大、市政协大力支持的结果，是全市上下齐心协力、共同奋斗的结果。在此，我代表市人民政府，向全体人大代表、政协委员，向工作在全市各条战线上的工人、农民、知识分子、广大干部、人民解放军指战员、武警官兵、政法干警以及各界人士致以崇高的敬意！向参与亳州建设的各位投资者和所有关心支持亳州发展的各界朋友，表示衷心的感谢！

各位代表，在肯定成绩的同时，我们也清醒地认识到，亳州经济社会发展中还存在不少困难和问题：一是经济总量偏小，综合实力不强，产业层次不高，自主创新能力较弱，水资源环境容量等要素约束加剧，保持经济又好又快发展难度增大。二是工业化进程缓慢，工业经济对全市经济的支撑作用仍然较低。三是城镇化水平低，城市功能和公共服务相对滞后。四是社会事业欠账较多，教育、卫生等社会事业存在一些短板，城乡发展不平衡，脱贫攻坚任务繁重艰巨。五是发展环境还不优，环境保护、安全生产、食品药品监管、征地拆迁工作还存在不少问题；少数干部为官不为、乱作为，一些领域和基层腐败现象还时有发生，政府工作水平和效率还有待进一步提高。对于以上问题，我们必须增强忧患意识、责任意识，勇于迎难而上、敢于攻坚克难，努力在发展中破解难题、寻求突破。

“十三五”时期的发展思路和目标任务

按照《中共亳州市委关于制定国民经济和社会发展第十三个五年规划的建议》，“十三五”时期我市经济社会发展的指导思想是：高举中国特色社会主义伟大旗帜，全面贯彻党的十八大和十八届三中、四中、五中全会精神，以马克思列宁主义、毛泽东思想、邓小平理论、“三个代表”重要思想、科学发展观为指导，深入贯彻习近平总书记系列重要讲话精神，认真落实“四个全面”战略布局，统筹推进“五位一体”建设，坚持发展第一要务，坚持创新发展、协调发展、绿色发展、开放发展、共享发展，坚持稳中求进、优中求快的总基调，以提高发展质量和效益为中心，以加快调结构转方式促升级为主抓手，以增进人民福祉、促进人的全面发展为出发点和落脚点，加快形成引领经济发展新常态的体制机制和发展方式，加快推进“三个亳州”现代化建设，确保如期全面建成小康社会。

主要目标是：到2020年，经济综合实力和竞争力走在皖北前列，地区生产总值达到1600亿元，人均地区生产总值超过5000美元，财政收入突破200亿元；转型升级实现突破，发展活力充分激发，城市功能明显增强，民生保障更加有力，文明程度不断提高，生态环境持续优化，社会大局保持稳定，与全国、全省同步实现全面建成小康社会宏伟目标。

（一）大力推进创新发展，构建现代产业新体系。实施产业集聚发展工程，确保战略性新兴产业产值占规模工业总产值比重达到35%以上，现代中药产业冲刺“双千亿”目标。推进传统产业改造升级和民营经济提质增效，力争工业化率达到40%以上。改造提升传统服务业，大力发展现代服务业，促进消费结构升级。调整优化农业结构，夯实现代农业基础，促进农业与二三产融合发展。建设一批研发中心，培育一批创新型企业，转化一批科技成果，研发一批创新产品。推进“互联网+”行动计划，拓展网络经济发展空间，全面提升经济社会信息化水平。构建创新发展体制机制，突出抓好行政体制、财税体制、金融创新、农业农村、国资国企等改革。

（二）大力推进协调发展，构筑城乡统筹新格局。走新型城镇化道路，全面提升城乡规划、建设和管理水平。完善重大基础设施建设和公共服务设施功能，增强城乡发展承载力。大力发展县域经济，充分发挥比较优势，培育壮大特色产业，增强县域经济发展综合实力。扎实推进美丽乡村建设。深入开展精神文明创建活动，力争创建全国文明城市。实施军民融合深度发展战略，持续增进军政军民团结。

（三）大力推进绿色发展，打造生态平衡新样板。促进人与自然和谐发展，构建科学合理的城市化格局、农业发展格局和生态安全格局。强化约束性指标管理，提高资源节约集约利用水平，加强集约节约用地，加大节能减排力度，建立循环型产业体系。加强环境综合整治，努力实现“凡土必绿、凡地必净、凡水必清、凡村必洁”目标。深入推进抓节水、保供水、治污水、防洪水、排涝水等工程。加强生态文明制度建设，严守资源环境生态红线，完善生态文明绩效评价考核体系。

（四）大力推进开放发展，拓展经济发展新空间。积极对接国家重大开放战略布局，深化对外交流合作，稳定外贸增长，优化营商环境。重点推进驻点招商、小分队招商、精准招商、借会招商、以商招商，力争利用市外内资年均增长15%左右、外商直接投资年均增长10%左右。推动省级开发区升格为国家级开发区，提高园区发展的质量和效益，力争全市园区生产总值比“十二五”末翻一番以上。

（五）大力推进共享发展，实现人民福祉新提升。实施脱贫攻坚工程，按照“三年集中攻坚，两年巩固提升”的总要求，坚持“五个一批”，落实“六个精准”，确保如期实现贫困人口全部脱贫、贫困村全部出列、贫困县区全部摘帽。办好人民满意的教育，全面普及15年基础教育，稳步提升学前教育三年毛入园率、九年义务教育完成率和高中阶段毛入学率。大力支持职业教育发展，培育高素质的技能型人才队伍。推进大众创业、万众创新，实现更高质量就业创业。努力提高城乡居民收入，拓宽居民收入增长渠道，城乡居民收入增速超过全省平均水平。加快推进多层次社会保障体系建设，织好民生安全保障网。推进养生亳州行动计划，满足人民群众多层次多样化健康需求。提高社会治理水平，促进社会更加和谐稳定。全面实施一对夫妇可生育两个孩子政策，促进人口均衡发展。

2016年的重点工作

今年是全面建成小康社会决胜阶段和“十三五”规划的开局之年，也是推进结构性改革、全面实施“调转促”行动计划的攻坚之年。全市经济社会发展的主要预期目标是：地区生产总值增长9%左右，财政收入增长10%左右，固定资产投资增长12%左右，规模工业增加值增长10%以上，社会消费品零售总额增长10%左右，进出口总额增长10%左右，城镇、农村常住居民人均可支配收入分别增长9%和10%左右，城镇登记失业率控制在4.2%以内，节能减排、大气和水污染防治完成省政府下达的年度目标任务。

各位代表，当前和今后一个时期，中央在适度扩大总需求的同时，着力加强供给侧结构性改革，提出宏观政策要稳、产业政策要准、微观政策要活、改革政策要实、社会政策要托底的五大政策要求，明确去产能、去库存、去杠杆、降成本、补短板五大重点任务。据此，我们将重点做好八个方面的工作：

（一）夯实经济稳定增长根基。

大力发展实体经济。全面开展“政企直通车”工作，建立手机终端微信服务平台，健全联系服务企业“找、交、销、查、考”工作机制，穷尽所能、倾尽全力、依法依规，切实解决企业发展中存在的实际问题。落实供给侧结构性改革政策，以降低实体经济企业成本为抓手，着力解决企业融资、用地、用工、用电、权证化、物流、税负以及制度性交易成本等占比偏高问题。力争新增规模以上工业企业100家、产值超亿元企业10家，新增个体工商户1.8万户、私营企业4000家。落实支持民营经济发展政策，鼓励民间资本进入基础设施建设、公共服务供给等领域。

着力扩大有效投资。全面落实项目建设“四督四保”制度，建成重点项目信息管理系统和项目服务微信平台，对全市年度计划实施的重点项目实行四级调度机制。充分发挥重大项目的牵头引领作用，全力配合商合杭客运专线建设，开工建设亳芜大道、银杏路下穿京九铁路立交桥和涡河高等级航道，积极推进亳蚌城际铁路、引江济淮入亳、亳州机场等项目前期工作。完成济祁高速公路利辛至淮南段主体工程，支持国省道路面改善工程建设，提高通行能力。密切跟踪国家、省重点投资领域和方向，精准谋划储备一批“大新专”和“调转促”项目，全年谋划储备项目1000个以上、投资总额不少于3000亿元。

大力培育新兴消费。加大停车场和新能源汽车充电设施建设力度，落实小排量汽车、新能源汽车税收优惠政策，扩大汽车消费。推动网络提速降费，加快形成线上线下联通互促的商业模式和消费方式，促进信息消费。推动旅游消费。大力发展养老家政健康消费。扩大教育文化体育消费。

（二）加快产业转型升级步伐。

集聚发展战略性新兴产业。围绕“双千亿”目标，实施“369”提升计划，以壮大企业规模、延伸产业链条、提升产业层次为核心，强力推进现代中药产业集聚发展基地建设。全市现代中药工业总产值增长20%以上、达280亿元，中药工业增加值占全市规模以上工业增加值的比重达25%，现代中药产业基地产值达267亿元，现代中药规模以上工业企业数达130家。大力发展农用机械装备、电子信息技术、新能源、节能环保等战略性新兴产业。

改造提升传统产业。紧紧依托我市农产品资源优势，开工建设市食品产业园，确保年内实现项目入驻。全年食品工业产值达330亿元，规模以上食品工业企业145家，年产值亿元以上加工企业60家，10亿元以上企业4家。改造提升白酒及饮料产业，积极支持古井集团、徽酒集团加大创新力度、提升品牌品质。提高煤炭资源开发和综合利用能力，支持涡阳煤矸石电厂建设，建成利辛电厂一期工程并实现并网发电。支持三县一区立足本地实际，推动食品及农副产品加工、纺织鞋服生产、汽车及零部件制造等产业加快发展。

着力推进园区建设。坚持组团式、特色化发展，按照产城融合的要求，推进亳芜现代产业园区、市经济开发区、安徽谯城经济开发区建设，配套完善教育、文化、卫生、体育等公共服务设施，优化功能布局，提升承载能力，加快转型升级。全市6个省级开发区实现产值230亿元，实现经营收入1030亿元，完成财政收入30亿元。

持续增强创新能力。充分发挥高新技术先导作用，加速培育壮大创新主体。新认定高新技术企业10家。发明专利授权量达140件以上。新增省级企业技术中心4家、省级新产品6个。新增省著名商标13件、市知名商标20件，培育申报中国驰名商标1件、国家地理标志商标1件。创新人才激励机制，加大人才培养和引进力度，为产业转型升级提供智力支撑。

（三）切实做好农业农村工作。

实施绿色增效转型工程。建设省级粮食绿色增产示范区（村、家庭农场）40个，优质专用型粮食生产基地达150万亩。粮食总产达96亿斤以上，全社会粮食收购130万吨。创建省级生态农业示范县1个、畜牧绿色低碳健康养殖示范场56个、渔业绿色健康养殖示范场4个，规模养殖比重达81%。中药材种植面积稳定在110万亩以上，其中标准化种植面积20%以上。蔬菜种植面积稳定在200万亩左右，其中设施蔬菜面积68万亩。培育壮大粮油、食品产业集群，规模以上农产品加工产值达650亿元。

健全现代农业经营体系。培育壮大新型农业经营主体，新创建市级以上示范合作社40家，新增家庭农场350家，重点培育8家合作社联合社。积极发展土地托管、土地流转等多种形式适度规模经营，适度规模经营面积达380万亩。大力发展农业产业化经营，培育现代生态农业产业化示范主体40个，创建市级以上农业产业化联合体29个。高度重视农产品加工销售，促进农业与二、三产业融合发展。推进农业品牌化建设，新增无公害农产品产地、绿色食品原料基地12个，新认证“三品一标”产品60个，着力打造一批有影响力的农业品牌。

夯实农业发展基础。继续推进农田水利基础设施建设，全年改造小型泵站1359千瓦，新建和加固小型水闸91座，改造中小灌区1.6万亩，扩挖塘坝1312座，清淤河沟655条，新打和修复机电井14700眼，改造末级渠系21万亩。推进农网升级改造，完成投资9.5亿元；加快建设220千伏利辛县夏湖输变电工程，开工建设500千伏涡阳县伯阳、220千伏谯城区华佗输变电工程。支持农机联合社重组，在国家补贴30%的基础上，市县两级再各叠加补贴15%鼓励农机购置，力争全市农机总动力达880万千瓦，主要农作物耕种收综合机械化率达88%以上，促进农业机械装备全面升级。建成市气象科普公园，建设气象防灾减灾标准化建设乡镇和示范村各8个。

继续开展农村土地综合整治。以开展建设用地增减挂钩工作为抓手，多争取用地指标。在原有基础上，认真抓好2016年城乡建设用地增减挂钩拆旧项目储备库建设，全年完成组卷1.5万亩，力争实施项目区拆迁复垦1万亩；抓好工矿废弃地复垦利用试点实施工作，复垦利用土地不少于5000亩；完成高标准基本农田建设任务100万亩，优先在贫困村实施；全年完成补充耕地1.5万亩。

（四）大力发展第三产业。

加快发展文化旅游业。完成北关历史街区改造规划。打造中心城区精品旅游线路。打通运兵道至人民北街南出口，推进白布大街等外立面修缮工程。完成道德中宫、曹操八角台遗址公园、江宁会馆修缮项目，开工建设观音山汉墓保护工程，谋划建设亳州市民俗非遗博物馆。支持涡阳县加快道源国家湿地公园、范蠡西施文化园项目建设，鼓励蒙城县统筹设计开发庄子祠、九鼎灵山寺旅游线路，推进利辛县西淝河休闲旅游、茨淮光谷·三十里白鹭源乡村旅游度假区建设。实施乡村旅游提升工程，培育乡村旅游精品线路3-5条。

推动金融业发展。持续加大银行业金融机构信贷投放，年末贷款余额增加160亿元以上。大力支持金融机构拓展表外融资业务。鼓励民间资本参股村镇银行和类金融机构增资扩股，积极引进域外银行、保险、证券机构入驻亳州。支持商业银行网点下沉，鼓励村镇银行增设网点。引导融资担保机构、小贷公司扩大资本金，做大比例再担保规模，设立新型农业经营主体贷款风险补偿基金。不断优化“我要贷款”平台，着力拓宽中小微企业融资渠道。支持企业加快上市挂牌步伐，推进广印堂、药都银行IPO进程，新增“新三板”挂牌企业10家、省股权托管交易中心挂牌企业10家。全面运行信用联合征信系统，坚决打击民间非法集资行为，持续优化金融生态环境。

着力转变流通方式。加快药通网、药博商城等平台建设，推进电子商务进农村试点工作，鼓励和支持企业在阿里巴巴、京东等电商平台开展网上营销。加快建设青年创客空间，帮助青年创业者开办电商企业。改造提升传统商贸流通业，力争全年培育新增限额以上商贸流通企业130家。新建高标准为农服务中心8家，新建和改造农家店160家。加快万达广场等重点商贸项目建设，打造一批特色商业街区。推进实施城区菜市场、水果和小吃摊点布点规划，加快建设乡镇商贸中心、商品配送中心和中心村连锁直营店。提高棚改、征迁货币化安置比重，加大进城农民购房贷款支持力度，精心调控商品房和商住用地供应量，保持房地产业继续平稳发展。

（五）持续释放改革开放红利。

推进各项重点改革。动态调整市、县、乡三级权力清单、责任清单和行政收费清单，通过有奖征询等方式健全完善公共服务清单。深入推进公共资源领域改革，整合建立全市统一的公共资源交易平台。深化商事制度改革，制订出台放宽住所登记管理办法和企业简易注销办法。继续深化财政体制改革，加强财政预决算管理，盘活财政存量资金。继续深化国企国资改革，建立健全以管资本为主的国资监管模式，支持古井集团、建安集团做大做强。继续深化农村综合改革，启动农村集体土地承包经营权和农民住房财产权抵押贷款试点工作，全面完成农村土地确权登记颁证工作，县区均建成农村产权交易中心。以发放“两证一书”为抓手，完成小型农田水利工程管理改革任务。推动重点领域价格改革，加强价格监管。

加快招商引资步伐。始终把招商引资作为第一要事，力争全年引进市外内资增长15%左右、达1380亿元。坚持把现代中药产业招商放在首位，把食品制造和劳动密集型产业作为重点，持续推进精准招商、以商招商和产业链招商，努力引进一批龙头企业和项目。坚持“抓大不放小”原则，在确保环保和投资强度的前提下，对成长性好、发展潜力大、投资额度在2000万元以上的中小项目予以准入，并严格约定项目开工、竣工时限。积极做好异地亳州商会建设工作，年内完成6家以上。落实财政扶持政策，吸引社会资本投资我市教育医疗、健康养老、文化旅游、体育健身等服务业。

大力发展外向型经济。重点抓好外贸进出口骨干企业的跟踪服务，力争全年新增孵化实绩企业20家。切实扩大外贸规模，大力支持中药提取物、养生保健品、农副产品深加工产品、劳动密集型产品和机电、高新技术产品出口，积极鼓励企业进口资源性产品，引进国外先进技术、设备。开展跨境电子商务。积极承接国际国内产业转移，结合我市主导产业发展，积极引进外资企业，全年外商直接投资增长10％左右。深入推进与央企、全国知名民企、外企对接，办好新一届药博会。推动企业“走出去”设立境外生产基地和营销机构，大力开拓国际市场。

（六）促进城乡协调发展。

准确把握“一尊重、五统筹”战略思想，以“九城同创”为总引领，坚持新区建设与老城改造齐头并进，坚持基础设施与公共服务供给同步提升，坚持市县乡村梯次推进、统筹联动发展，大力推进以人为核心的新型城镇化。

提升中心城区功能。坚持“规划一张图，城乡全覆盖”，启动编制市全域空间统筹规划、新型城镇化试点规划，编制完成地下空间综合利用规划。突出抓好路网、水网、地下管网等公共基础设施和服务设施建设，道西片区全面实现“九通一平”。年内市中心城区实施城建新建项目96个、续建项目167个，完成投资160亿元。新改建道路17条、22公里；开工建设亳州南公路公交客运高铁枢纽站和亳州北公路公交客运枢纽站；完成市城区4个老站综合改造利用，新建、改造魏武广场等大型停车场5个；优化公交线路3条。支持谯城区重点抓好北部新城建设，加快华佗广场、郑店子、筛子市等棚户区还原房建设步伐。

加快建设三县县城。深入推进县城“多规合一”试点，提升规划设计水平。支持涡阳县加快南部新区建设，建设德铭、华都等安置小区，建设德昌路、育英路等城市道路。支持蒙城县实施仁和路等20条道路配套建设工程，建设南华苑小区等保障房和棚改项目，改造老城区小街背巷115条。支持利辛县实施建设南路、人民北路等建设工程，抓好阜蒙河等沟河综合整治，改造老城区污水管网。

持续抓好美丽乡村建设。抓紧编制“十三五”时期美丽乡村建设专项规划，进一步优化中心村布点规划，认真修编乡镇建设规划，编制中心村建设规划。全面建成2015年度31个省级重点美丽乡村。完成2016年度37个乡镇政府驻地整治建设任务，突出抓好治脏、治乱，加强基础设施建设和公共服务配套；同步推进其所在行政村省级中心村建设。完成所有行政村村庄布点规划，并启动一期占地至少10亩的新村建设。扎实做好利辛县整县推进美丽乡村建设试点工作。各县区每年重点建设2-3个美丽集镇，突出抓好各县区集镇卫生、交通、建筑、经营四大秩序整治工作。深入开展农村清洁工程、“三线三边”治理和“三治三增三提升”行动，力争创建省级生态乡镇4个、省级生态村5个。

（七）着力提升生态环境质量。

着力解决环境突出问题。更加注重精细管理，全面实施数字城管，完善城市管理同步指挥平台，把老城区、小街巷、老旧小区全部纳入数字城管系统，完善工作机制，切实解决环境脏乱差、管理不规范、责任不到位等问题。加强工业、生活节水宣传，推广中水回用，抓好节约用水。实施城区供水管网新改建工程，大幅降低漏水率，加强水质监测，推进深层地下水采水置换，提高供水质量，满足人民生产生活用水需求。重点做好“一涵四闸”范围内河流截污和丁家坑、鳖疙瘩坑黑臭水体整治工作，启动市污水处理厂、涡北污水处理厂和南部新区污水处理厂扩建工程，日污水处理能力由12.5万吨提高到22万吨，逐步解决城区水环境污染问题。积极开展海绵城市创建工作，着力解决宋汤河、凤尾沟、涡河城区段、陵西湖等处城市内涝及雨水收集问题。实施路灯改造工程，建成区亮灯率达到98%。完成60家规模化畜禽养殖场污染综合治理任务，减少农业面源污染。大力推行环境监管网格化管理，落实生态环境损害责任终身追究制度。

继续实施“地净、路畅、水活、天蓝、林拥城”工程。加强环卫基础设施建设，提升洒水车、清扫车、垃圾运输车、渣土车、混凝土搅拌车信息化监管水平。开工人民西路、汤王大道北段等20条“断头路”延伸工程和南、西、北外环快速通道建设，启动建设建安路涡河隧道和汤王大道涡河隧道，完成占道小商品售卖亭整治工作。启动建设宋汤河五期、凤尾沟二期工程，全面完成“一涵四闸”项目建设。强化工业废气、汽车尾气、施工扬尘、城市道路保洁等专项治理，加强对全市83家市控以上企业的环境监管，完成35家重点企业在线监测视频监控，确保达标排放；在市主城区全面推行50客座以上餐饮单位安装油烟净化设施，统筹抓好黄标车治理、秸秆禁烧、垃圾焚烧、油气回收等工作，PM10平均浓度下降8个百分点。建成高标准环城林带和市植物园。持续推进“增绿增彩增果”活动，认真实施千万亩森林增长工程，完成人工造林8.4万亩，建设绿色长廊480公里。扎实开展国家园林城市创建工作，建设城市绿道20公里。

抓好资源节约集约利用。强化约束性指标管理，实行能源和水资源消耗、建设用地等总量和强度双控行动。坚持节约集约用地，优化建设用地结构，推进低效用地再开发。全面开展园区用地情况调查摸底工作，加大闲置低效用地清理力度，创新闲置土地盘活方式，建立项目退出机制，建成标准化厂房40万平方米。实施全面节能行动计划，强化用能重点领域和单位节能管理。积极发展太阳能、生物质能等清洁能源，建成市生活垃圾焚烧发电厂，利辛县信利新能源100兆瓦光伏电站、谯城区古井镇生态农场40兆瓦光伏电站并网发电。

（八）倾力保障和改善民生。

实施脱贫攻坚工程。坚持把脱贫攻坚作为第一民生工程，以扶贫开发“四项清单”为主抓手，落实“六个精准”要求，重点实施“八大工程”，实现贫困村出列88个、脱贫10.4万人。大力发展光伏扶贫，集中建设1.2万户、95个村光伏发电项目。改造3000户农村贫困户危房，完成286个建档立卡贫困村道路硬化、电网改造和4G网络覆盖工程。全部免除贫困家庭高中学生学杂费。实行脱贫工作责任制，建立健全定点扶贫帮扶长效机制，全面推行“单位包村、干部包户”和驻村工作队制度，确保每个贫困村都有驻村工作队、每个非贫困村都有1名扶贫专干、每个贫困户都有1名帮扶联系人，实行逐户销号，做到脱贫到人。

优先发展教育事业。明确县区教育主管部门管理职责。全面推行中小学教师无校籍管理，加大城乡教师交流力度。加强校长队伍建设。实施中心城区中小学幼儿园布点规划，年内新建中小学7所、改扩建小学2所，建设公办幼儿园7所、投入使用5所。深入推进义务教育薄弱学校改造和千所村小提升工程，实现村小建设与新村布点规划相衔接。以推广远程教育为抓手，提升教育教学质量。持续做好国学经典教育、书香校园建设工作。实施职业教育提升计划，加快发展现代职业教育，推进产教融合、工学结合、校企合作，支持亳州职业技术学院创建地方技能型高水平大学，继续做好亳州师专、亳州幼师、亳州中药科技学校提升创建工作。开展对贫困家庭子女、未升学初高中毕业生、农民工、失业人员和转岗职工、退役军人免费培训工作。开工建设市特教学校新校区。

大力发展卫生计生事业。深入推进养生亳州行动计划。继续探索医药卫生制度综合改革，实行医疗、医保、医药联动。加大社会办医力度，年内开工建设二级及以上医院10家。建成市医院新院二期工程，完成华佗中医院新院主体工程，谯城区人民医院、市精神病医院投入使用，加快市精神病福利中心建设。提升乡镇卫生院、社区卫生服务中心中医药服务能力。加强乡镇卫生院、乡村医疗队伍建设，安排每个乡镇卫生院至少招考1名本科医学毕业生，纳入编制管理，财政全额供给。全面完成县、乡两级妇幼保健和计划生育服务机构整合工作。提高计生特扶对象中失独家庭扶助标准，在原有扶助金基础上每人每月增加1000元。全面实施一对夫妇可生育两个孩子政策。人口自然增长率控制在9.5‰以内。

提升公共文化体育服务能力。推进公共文化服务设施建设，完成市五位一体公共活动中心建设主体工程，建成乡镇（街道、社区）综合文化服务中心4个、农民文化乐园10个。加快“三网融合”步伐，亳州广播电视台三个电视频道信号覆盖受众新增10万户以上；继续实施广播“村村通”工程，亳州广播电视台两个广播频率信号覆盖到全市自然村，市、县广播电视台每天播放自办广播节目2小时以上。开工建设市广电中心。广泛开展送戏送电影下乡、送戏到敬老院活动，做到每个行政村和敬老院1年分别至少两场文艺演出、1村1月1场电影。支持民营文化企业发展壮大。举办市第二届运动会，建设城市社区15分钟健身圈，大力推广普及五禽戏。

全力做好就业和社会保障工作。坚持以创新促进创业、以创业带动就业，采取多种形式建设农民工、大学生创业孵化基地和青年创业园，新增城镇就业3万人，新增转移农村富余劳动力7.2万人。继续做好省安排的民生工程各项工作。健全社会保障体系，稳妥推进机关事业单位养老保险制度改革，开展社会保险扩面征缴工作。持续加强社会救助工作，落实临时救助制度，完善重特大疾病医疗救助政策，加大孤儿、艾滋病人、精神病人、长期卧床病人帮扶关爱力度，着力保障困难群体基本生活，城市、农村低保标准分别提高10%和25%以上，五保供养标准平均提高10%以上。新开工保障性安居工程25056套，基本建成20833套，力争棚户区改造货币化安置比例达50%。

积极创新社会治理。加强精神文明建设，弘扬社会主义核心价值观，传承优秀传统文化，扎实推进书香亳州建设，深入开展中国好人、道德模范评选和宣传活动，提高市民素质和社会文明程度，营造向上向善的社会风尚。启动“七五”普法工作，推进公共法律服务体系网络平台和实体平台建设。安排全市每个行政村每年5万元办公经费列入财政预算，解决有钱办事问题。不断完善社会治安防控体系，扎实推进“平安亳州”建设，全面建成中心城区、三县县城、谯城区农村公共区域视频数据平台和社会管理服务系统，坚决预防和打击各类违法犯罪活动。大力推进食品药品安全城市创建，着力加强食品药品生产经营各环节专项整治，突出抓好农贸市场、小餐饮、小作坊、零售药房等生产经营场所达标规范工作，切实保障人民群众饮食用药安全。认真落实“分管市（县）领导+主管部门+属地”三位一体责任制和领导干部开门接访工作制度，依法做好初信初访、诉访分离和信访积案化解工作，推进信访工作信息化，打造阳光信访、责任信访、法治信访。严格落实“党政同责、一岗双责、失职追责”，持续加大安全隐患排查整改力度，当年整改率达98%以上。

推进军民融合深度发展，广泛开展全民国防教育，不断加强民兵预备役建设，支持武警部队基地建设。提升消防装备水平，开工建设芍花路、人民路消防站。做好民族、宗教工作。抓好关心下一代工作。关爱留守儿童、留守妇女和留守老人。开展第三次全国农业普查工作。支持残疾人事业发展。加强外事、侨务、对台、防震减灾、应急管理、无线电和机关事务管理工作，推动档案、地方志、红十字事业健康发展。

进一步加强政府自身建设

适应新常态，推动新发展，满足新期盼，对政府自身建设提出了新的更高要求。我们要自觉践行“三严三实”，强化为民、为企服务，转变政府职能，改进工作作风，提升政府公信力和执行力。

全面坚持依法行政。加强法治政府建设，把依法行政贯穿政府工作始终，严格规范政府行为，确保一切工作在法律框架内运行。坚决维护市委领导，自觉接受人大及其常委会的法律监督、工作监督和政协的民主监督，认真办理人大代表议案、建议和政协委员提案。完善政府协商机制，加强与人大代表、政协委员以及民主党派、无党派人士、工商联等的沟通协商，重视听取工会、共青团、妇联等人民团体以及社会各方面的意见和建议。坚持科学决策、民主决策、依法决策，健全重大决策终身责任追究制度及责任倒查机制。全面推行政府法律顾问制度，扎实做好行政复议工作。坚持公正文明执法，重点规范行政许可、行政处罚、行政强制、行政收费、行政检查等行为。大力推行政务公开，继续办好行风热线、“沟通——政务零距离”等问政栏目，主动接受新闻媒体和社会公众监督。

全面强化服务意识。牢固树立服务导向，紧紧围绕人民群众关心的热点问题，紧紧围绕社会各界反映强烈的焦点问题，紧紧围绕制约亳州长远发展的难点问题，勤听意见，勤谋对策，勤抓落实。建立健全服务机制，持续不断下基层、走企业、访群众，畅通“12345，电话找政府”市长热线、市民论坛、市长信箱等服务渠道，提升网上办事大厅和“我家亳州”、“政企直通车”等平台功能，努力为基层、为企业、为群众解难题、办实事。

全面改进工作作风。坚持问题导向，千方百计查找工作中存在的问题，实行台账管理、办结销号和季度通报制度，不回避、不延误，扎实有效地解决问题。认真制定年度岗位责任目标，全面强化督查考核，抓全年、全年抓，积极作为，奋发有为，一抓到底、抓出成效，确保各项工作落地有声。加强政府效能监察，加大行政问责力度，确保政令畅通。始终保持抓铁有痕、踏石留印的狠劲，始终保持咬定青山不放松的韧劲，快字当头，实处发力，提高工作绩效。全体政府系统干部要保持蓬勃朝气、昂扬锐气，共产党员要做“坚定不移向党中央看齐，奋力争当‘四个自觉’模范”的标兵。

全面加强廉政建设。坚持清廉从政、从严治政，认真履行党风廉政建设“一岗双责”，大力推进惩防体系建设，建立完善长效机制。始终保持反腐高压态势，强化权力运行制约监督，严肃查处各类违纪违法案件，切实解决以权谋私、损害群众利益的问题。按照审计监督全覆盖的要求，加大政策措施落实、公共财政资金、政府投资项目、经济责任履行等审计力度，注重审计结果运用，充分发挥审计监督作用。厉行勤俭节约，反对铺张浪费，严防“四风”反弹，严控“三公”支出，树立为民、务实、清廉的良好形象。

各位代表！“十三五”的美好蓝图已经绘就，2016年的工作重任在肩。让我们在中共亳州市委的坚强领导下，勠力同心，锐意进取，崇尚实干，狠抓落实，为加快推进活力亳州、美丽亳州、幸福亳州现代化建设而努力奋斗！
